# Onset of Multiple Chronic Conditions and Depressive Symptoms: A Life Events Perspective

**DOI:** 10.1093/geroni/igx022

**Published:** 2017-11-17

**Authors:** Maureen Wilson-Genderson, Allison R Heid, Rachel Pruchno

**Affiliations:** 1 Temple University School of Public Health, Philadelphia, Pennsylvania; 2 New Jersey Institute for Successful Aging, Rowan University School of Osteopathic Medicine, Stratford

**Keywords:** Depressive symptoms, Multimorbidity, Multiple chronic conditions

## Abstract

**Background:**

While the association between depressive symptoms and chronic illness has been the subject of many studies, little is known about whether depressive symptoms differ as a function of the illnesses people have as they transition to living with multiple chronic conditions.

**Methods:**

Self-reports of five diagnosed chronic conditions (arthritis, diabetes, heart disease, hypertension, and pulmonary disease) and depressive symptoms were provided by 3,396 people participating in three waves of the ORANJ BOWL^SM^ research panel. Longitudinal multilevel modeling was used to examine the effects that transitioning to having a diagnosis of multiple chronic conditions has on depressive symptoms.

**Results:**

Between 2006 and 2014, controlling for age, gender, income, race, and a lifetime diagnosis of depression, people who transitioned to having a diagnosis of multiple chronic conditions had significantly higher levels of depressive symptoms than people who did not make this transition. The diagnosis of arthritis, diabetes, heart disease, and pulmonary disease, but not hypertension had independent effects, increasing depressive symptoms.

**Conclusions:**

Having a diagnosis of multiple chronic conditions leads to increases in depressive symptoms, but not all illnesses have the same effect. Findings highlight the need for clinicians to be aware of mental health risks in patients diagnosed with multiple chronic conditions, particularly those with a diagnosis of arthritis, diabetes, heart disease, and pulmonary disease. Clinical care providers should take account of these findings, encouraging psychosocial supports for older adults who develop multiple chronic conditions to minimize the negative psychological impact of illness diagnosis.

**Translational Significance**

The results suggest the need for clinicians to be aware of mental health risks in patients diagnosed with multiple chronic conditions, particularly those with a diagnosis of arthritis, diabetes, heart disease, and pulmonary disease. Clinical care providers should encourage psychosocial supports for older adults who develop multiple chronic conditions to minimize the negative psychological impact of illness diagnosis.

Declining mortality rates and an aging population have led to an increase in the number of people living with two or more chronic conditions (Salive, 2013). Nearly 70% of older Americans (age 65+) have multiple chronic conditions (MCCs)—two or more diseases that are long in duration and devoid of spontaneous resolution (Held et al., 2015; Rocca et al., 2014)—with 24.7% having 3 or more, and 11.5% having four or more (Laditka & Laditka, 2016). The Institute of Medicine (Institute of Medicine, 2012) estimates that by 2030, 37 million baby boomers will have MCCs. Rates of MCCs are similar in other developed nations (Barnett et al., 2012); globally, multimorbidity patterns in low- and middle-income countries parallel those of wealthier countries (Garin et al., 2015). Medical costs for people with MCCs represent 75% of the U.S.’ $2 trillion annual health care budget (Kaiser Family Foundation, 2015); similar economic impacts are seen in other countries (Abegunde, Mathers, Adam, Ortegon, & Strong, 2007). MCCs increase the inpatient costs of hospital care because they result in longer lengths of stay (Skinner, Coffey, Jones, Heslin, & Moy, 2016).

Until recently, chronic illnesses such as cancer, diabetes, and heart disease were predominantly studied in silos, although some studies focused on a single “index” condition, treating other conditions as secondary (Vos, van den Akker, Boesten, Robertson, & Metsemakers, 2015). Recognizing that older people often experience chronic conditions in conjunction with one another, the U.S. Department of Health and Human Services (Services, 2010) called for a paradigm shift from the study of individual chronic conditions to the study of MCCs, and studies examining dyads and triads of illnesses provide evidence of additive, subtractive, and synergistic effects of MCCs on quality of life (Galenkamp, Braam, Huisman, & Deeg, 2011; Garin et al., 2015; Hunger et al., 2011; McDaid et al., 2013; Ralph, Mielenz, Parton, Flatley, & Thorpe, 2013; Stenholm et al., 2015; Tinetti et al., 2011). However, while research has begun to address the negative raminifactions of living with MCCs, little is known about how the transition from not having MCCs to having MCCs affects individual well-being. The purpose of this study is to explore the impact of transitioning to MCCs on depressive symptoms in older adults.

## MCCs and Depression

Of particular concern, clinically diagnosable depression is two to three times higher among people with any chronic illness compared to individuals without chronic illness and may be as high as 25% among people with MCCs (Katon, 2011). Yet, the causal association between chronic physical illnesses and depression remains unclear. Using data from the Health and Retirement Study, Karakus and Patton (2011) found that older people with depression had a significantly greater risk of developing diabetes, heart problems, and arthritis, but not cancer, during a 12-year follow-up. Furthermore, a meta-analysis of longitudinal studies by Rotella and Mannucci (2013a) found that depressive symptoms are associated with a significantly increased risk of incident diabetes, while another meta-analysis of longitudinal studies by Rotella and Manucci (2013b) found that people with diabetes were at an increased risk for depressive symptoms. Other research focused on single-illnesses indicates that the risk of developing depressive symptoms increases after a new diagnosis of cancer, diabetes, hypertension, heart disease, arthritis, chronic lung disease, or stroke (Huang, Dong, Lu, Yue, & Liu, 2010; Polsky et al., 2005). Additional prior work demonstrates that while chronic illnesses are associated with increased depressive symptoms, most of these effects diminish with age, so that people who develop chronic illnesses earlier in life report more depressive symptoms than people who develop chronic illness later in life (Schnittker, 2005). Still other studies provide evidence for reciprocal or bidirectional associations between chronic illnesses and depressive symptoms (Chen, Chan, Chen, Ko, & Li, 2013; Golden et al., 2008; Liew, 2011).

Pruchno, Wilson-Genderson, and Heid (2016) found that controlling for demographics and the number of chronic illnesses, the composition of illnesses affected level of depressive symptoms. For example, among people having only two of five chronic conditions, those with arthritis-pulmonary disease had significantly higher depressive symptom scores than people with heart disease-hypertension or diabetes-hypertension. Among people with three chronic conditions, those with arthritis-heart disease-pulmonary disease had significantly higher depressive symptom scores than people with arthritis-heart disease-hypertension, arthritis-hypertension-pulmonary disease, arthritis-diabetes-hypertension, or diabetes-heart disease-hypertension.

These findings highlight the impact of the presence of chronic illness on depressive symptoms. However, they raise important questions about the association between the process of transitioning to having MCCs (i.e., the onset of becoming a person with MCCs) and depressive symptoms. It is possible that among people without chronic illnesses or those with a single chronic illness, the diagnosis of additional chronic illnesses leads to higher levels of depressive symptoms. But, it is also possible that the diagnosis of only certain illnesses results in more depressive symptoms. This issue has important clinical implications, because preventive interventions designed to limit depressive symptoms can be better targeted.

## Conceptual Framework

Diagnosis of an individual chronic illness has been conceptualized as a life stressor—an event that has the potential to change the expectations the person has for him/herself and the way he/she is perceived by other people (Aneshensel, 1992; Bury, 1982). Diagnosis may cause the person to make changes in health behaviors such as diet, smoking, and exercise and may necessitate changes in daily routines (Newman, Steed, & Mulligan, 2004). Diagnosis may require embracing a complex medication regimen, and may force the person to confront his/her mortality. Increases in depressive symptoms following a single diagnosis are common (Deimling, Kahana, Bowman, & Schaefer, 2002; Hollingshaus & Utz, 2012; Massie, 2004; Polsky et al., 2005; Schnittker, 2005; Stanton, Revenson, & Tennen, 2007).

Within the context of theories about stressful life events (Holmes & Rahe, 1967; Zautra, Schultz, & Reich, 2000), it stands to reason that being diagnosed with multiple chronic conditions should be stressful and should be associated with an increase in depressive symptoms. The event of MCC diagnosis may result in a cascade of challenges, such as loss of independence and control over one’s life or organic disease-based decline in cognition, both of which may lead to depression (Williamson, Shaffer, & Parmelee, 2000). Cross-sectional studies consistently find that people with more chronic illnesses experience higher levels of depressive symptoms (Murrell, Himmelfarb, & Wright, 1983) and lower quality of life (Fortin, Dubois, Hudon, Soubhi, & Almirall, 2007; Heyworth, Hazell, Linehan, & Frank, 2009). However, Galenkamp and colleagues (2011) found that the association between the number of chronic diseases and self-reported health was nonlinear, except among the oldest old, and longitudinal studies find evidence that depressive symptoms dissipate as time since diagnosis increases (Polsky et al., 2005; Stommel, Kurtz, Kurtz, Given, & Given, 2004). Furthermore, because chronic illnesses usually are diagnosed sequentially, at different points in a person’s life, it could also be that people adapt to each illness as it unfolds, rendering the transition to having multiple chronic conditions no more stressful than diagnosis of a single condition.

In one of the few studies to examine the depressive symptom trajectories associated with different chronic conditions, Chiu, Hsu, and Tseng (2017) found four patterns of association: (1) elevated depressive symptoms that worsened over time after a diagnosis of heart disease, arthritis, or hypertension, (2) elevated depressive symptoms did not worsen over time after a diagnosis of stroke, lung disease, gastric conditions, or liver disease, (3) no elevated depressive symptoms after diagnosis but an increase in depressive symptoms over time for people with diabetes, and (4) no significant associations between depressive symptoms and diagnosis of cancer. As a result, further study of the impact of MCC onset on depressive symptoms is needed.

## Present Study

This article uniquely explores the role of onset of MCCs. The goal of our analyses is to examine whether the transition from not having to having MCCs results in an increase in depressive symptoms and whether the diagnosis of specific illnesses, including arthritis, diabetes, heart disease, hypertension, and pulmonary disease, has significant, independent effects on depressive symptoms. We also recognize that depressive symptoms result from a complex interplay of factors acting over the lifetime of an individual and analyses control for demographic characteristics including age, gender, income, and race. Additionally, because it is possible that people who had been diagnosed with depression earlier in life may have different patterns of change in depressive symptoms, we control for lifetime diagnosis of depression.

## Methods

### Participants

Baseline (T1) data from 5,688 people participating in the ORANJ BOWL panel (“*O*ngoing *R*esearch on *A*ging in *N*ew *J*ersey: *B*ettering *O*pportunities for *W*ellness in *L*ife”) were collected by telephone interviews between 2006 and 2008. The goal of the ORANJ BOWL panel is to understand factors that influence successful aging. Participants were recruited by cold calling using list-assisted random-digit-dialing (RDD) procedures. Eligible people were between the ages of 50 and 74, living in New Jersey, and had the ability to participate in a 1-hr, English-language telephone interview. Demographics of the targeted sample made coverage loss due to cell phone-only households very small (Blumberg & Luke, 2007). Using standard American Association for Public Opinion Research calculations, ORANJ BOWL achieved a response rate of 58.73% and a Cooperation Rate of 72.88%, consistent with or better than average RDD response rates. Details regarding sample development are provided in Pruchno, Wilson-Genderson, and Cartwright (2010). The sample was representative of older adults (aged 50–74) living in New Jersey in 2006 in terms of race (Caucasians/Blacks). Hispanics were under-represented in the sample because the study lacked funds for interview translation (Pruchno et al., 2010). As is true for other studies, ORANJ BOWL, had a slightly higher rate of participation by women and individuals with more years of education (Pruchno et al., 2010).

The T1 ORANJ BOWL sample included 2,067 men and 3,621 women, who had a mean age of 60.7 (*SD* = 7.1). The modal education level among participants was high school graduate (28.3%). The majority of respondents were White (83.8%); 11.8% were African American. The majority of respondents were currently married (56.7%) with a mean household income between $30,000 and $80,000 (29.8%), whereby 19.1% reported less than $30,000 and 41.1% reported more than $80,000. ORANJ BOWL participants were well dispersed throughout the state, residing in 1,644 of New Jersey’s 1,912 census tracts (Cromley, Wilson-Genderson, Christman, & Pruchno, 2015).

A subsample of participants was recontacted one year after their T1 interview and asked to complete a personality measure (T2). Because questions about chronic health conditions were not included at this wave, these data were excluded from the present analysis.

**Figure 1.**
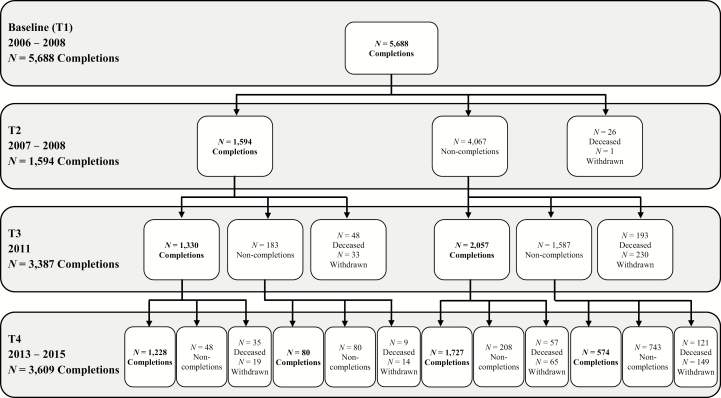
Study sample flow.

In 2011, a questionnaire (T3) was mailed to all ORANJ BOWL respondents (see Figure 1 for sample flow). People completing the questionnaire were younger (*F* = 53.78, *df* = 2; 5,685), less likely to be African American (*F* = 43.17, *df* = 2; 5,685), had more years of education (*F* = 73.15, *df* = 2; 5,672), wealthier (*F* = 79.87, *df* = 2; 5,019), and more likely to have been married at T1 (*F* = 29.46, *df* = 2; 5,678) than those who died or did not complete the T3 questionnaire. Completers were also more likely to be women than those who died (*F* = 4.69, *df* = 2; 5,685; i.e., proportionally more men died).

In 2014, a questionnaire (T4) was mailed to all ORANJ BOWL respondents known to be alive at T3 (see Figure 1 for sample flow). People completing the T4 questionnaire were less likely to be African American (*F* = 44.41, *df* = 2; 5,685), more likely to have greater years of education (*F* = 98.70, *df* = 2; 5,672), more likely to be wealthier (*F* = 137.38, *df* = 2; 5,019), and more likely to have been married at T1 (*F* = 57.71, *df* = 2; 5,678) than those who had died or did not complete the T4 questionnaire. Completers were also more likely to be female (*F* = 5.37, *df* = 2; 5,685) and younger (*F* = 96.34, *df* = 2; 5,685) than those who died since T1, but were older than noncompleters. The T4 sample size is bigger than T3 due to the availability of funds for additional sample follow-up which were not available at T3. Overall, attrition differences from one wave to the next may result in an underrepresentation of individuals of lower education, lower income, and/or of minority status; results should be interpreted with this potential bias in mind.

### Measures

#### Chronic conditions

ORANJ BOWL^sm^ participants reported at each wave whether or not a physician or health care professional had ever told them they had any of 14 health conditions by responding to questions drawn from the National Health Interview Survey. Five chronic conditions were selected for the analyses that follow: arthritis, diabetes, heart disease, hypertension, and pulmonary disease. These conditions were selected because they are (1) among the 20 selected by the U.S. Department of Health and Human Services Office of the Assistant Secretary of Health as part of a strategic framework for improving the nation’s response to the challenge of multimorbidity (Goodman, Posner, Huang, Parekh, & Koh, 2013); (2) they are among the most prevalent diseases of US adults (Ward, Schiller, & Goodman, 2014); (3) they are among the most common causes of death and diminish quality of life (Institute of Medicine, 2012); and (4) they are each long in duration and void of recovery, requiring continual monitoring and self-management behaviors on the part of the patient. Regarding this last criterion, for example, we did not include cancer due to the fact that cancer can be treated and a person can become cancer free. While an individual may need to undergo periodic medical monitoring, the everyday self-management behaviors for cancer diminish after active treatment.

At baseline, just over a quarter of the sample (*n* = 1559, 27.4%) reported none of the five health conditions, 32.3% (*n* = 1837) reported one condition, 22.5% (*n* = 1279) reported two conditions, 12.4% (*n* = 709) reported three conditions, 4.3% (*n* = 244) reported four conditions, and 1.0% (*n* = 60) reported all five conditions. The most prevalent condition was hypertension (46.6%), followed by arthritis (40.2%), pulmonary disease (18.6%), heart disease (16.1%), and diabetes (15.7%). Additional details can be found in Pruchno et al. (2016). As the goal of these analyses was to examine onset of multiple chronic conditions (transitioning from zero or one condition to two or more conditions), the sample used in the present analysis was restricted to participants with zero or one diagnosed chronic condition (“onset sample”) at baseline (*n* = 3,396). At each wave subsequent to baseline (T3 or T4) participants were asked if a diagnosis of each of these five illnesses had been made. Onset of a condition between waves was defined as not having the condition at the prior wave, but reporting a diagnosis at the next wave. A comparison of the excluded sample (*n* = 2,290) and the onset sample revealed that the onset sample was younger (61.2 vs 67.3; *t* = 19.5, *p* < .0001), less likely to be female (61.2% vs 67.3%; χ^2^ (1) = 21.9, *p* < .0001), had higher income (4.42 vs 3.47; *t* = 18.75, *p* < .0001), and less likely to be African American (8.3% vs 15.8%; χ^2^ (1) =76.9, *p* < .0001). The onset sample was also less likely to have a lifetime diagnosis of depression (19.7% vs 30.0%; χ^2^ (1) = 79.1, *p* < .0001) and to have died during the course of this study (6.7% vs 18.1%; χ^2^ (1) = 176.5, *p* < .0001).

To capture the potential influence of the diagnosis of a new chronic condition on depressive symptoms, a time-varying predictor that specified whether the event in question occurred (diagnosis of any individual condition) was created for each illness. The event of each individual disease diagnosis was used to indicate when a participant moved from non-diagnosed (time-varying predictor = 0) to diagnosed (time-varying predictor = 1). Thus, a participant who was newly diagnosed with hypertension between T3 and T4 would have the time-varying covariate for hypertension of 0 at baseline and T3 and 1 at T4. Participants who had any one condition at baseline (i.e., hypertension) were not included as participants experiencing onset during the frame of this study; we control for baseline status to account for this. A separate dummy variable (Onset MCC) was created to capture the variance attributable to becoming MCC or remaining free of multiple chronic conditions (zero or one chronic condition) at T4. The time-varying predictors for onset of individual chronic conditions, as well as the MCC onset dummy variable, were included in the final model.

### Depressive Symptoms

Depressive symptoms were measured at each wave using the 10-item short form of the Center for Epidemiologic Studies Depression scale (CES-D; (Andresen, Malmgren, Carter, & Patrick, 1994). Each item was scored from 0 (*none of the time*) to 3 (*most of the time*). Two items (“I felt hopeful about the future” and “I was happy”) were reverse coded. Total scores ranged from 0 to 30; Chronbach’s α = .82. Higher scores indicate more depressive symptoms.

### Lifetime Diagnosed Depression

Participants reported at baseline whether or not a physician had ever told them they had depression, anxiety, or another emotional problem; this was coded as 0 (*no*) or 1 (*yes*). A quarter of the sample reported having had a lifetime diagnosis of depression (*n* =1,357).

### Individual Characteristics

At baseline, respondents reported their age, gender (0 = *male*, 1 = *female*), income (range from 1 = *less than $15,000* to 6 = *more than $150,000*), and race (0 = *not African American*, 1 = *African American*).

### Statistical Analysis

Multilevel mixed effects models, which account for the nesting of observations within participants, were developed to examine the association between the diagnosis of new chronic conditions and depressive symptoms. These models permit the use of all available waves of data for all participants and produce unbiased estimates in the presence of missing data. A within-person unconditional model (*Y*_*it*_ = *ð*_*0i*_ + *ð*_*1i*_ (*wave*_*it*_) + *e*_*it*_) was created to estimate average linear and person-specific change over time in depressive symptoms, where *Y*_*it*_ is the CES-D score at time *t* for participant *i*; *ð*_*0i*_ is initial status of CES-D for participant *i.* The linear time parameter *ð*_*1i*_ is coded as 1, 3, 4 (wave) and represents the point of measurement for the linear change in depressive symptoms and *e*_*it*_ is the error for participant *i* at time *t*. Once the form of the change and average slopes were determined, additional variables were introduced as follows: Model B added demographic characteristics (age, gender, income, and race) to account for associations between person-level characteristics and depressive symptoms; Model C added prior lifetime diagnosed depression; Model D added a dummy variable reflecting transition to having MCCs (0 = no transition; 1 = transition); and Model E added information about the diagnosis of arthritis, hypertension, heart disease, diabetes, and pulmonary disease (0 = no diagnosis; 1 =diagnosis). Model results are presented as regression parameter estimates (β) with the associated standard errors, and the significance test assessed as *p* values less than .05. Fit indices are presented for sake of model comparison. A computed Cohen’s D effect size for significant effects in the final model are reported in text. In order to account for risk of death, sensitivity analyses were conducted following procedures described by Berry, Ngo, Samelson, and Kiel (2010). Although people who died were more depressed than those who did not, death status did not affect other findings. Statistical analysis was conducted using SAS 9.4.

## Results

The sample had a similar age and gender distribution across waves of data collection; however, the racial composition became more homogenous (Table 1). The most prevalent chronic illness at baseline was hypertension followed by arthritis. Over the course of eight years, 915 individuals reported a diagnosis of arthritis (16%); 731 reported a diagnosis of hypertension (13%); 260 reported a diagnosis of pulmonary disease (5%); 198 reported a diagnosis of heart disease (3%); and 149 reported a diagnosis of diabetes (3%). Of the participants for whom there was T4 data, 1,323 who at baseline had no or one chronic illness remained at zero or one chronic condition at T4; 944 people transitioned to having MCCs at T3 or T4.

**Table 1.** Sample Descriptives and MCC by Time

	T1 *N* = 3,396	T3 *N* = 2,112	T4 *N* = 2,197
Age (*M* [*SD*])	59.3 (6.8)	64.35 (6.7)	66.4 (6.7)
Sex (Female)	2,080 (61.2)	1,318 (62.4)	1,361 (61.9)
African American	283 (8.3)	117 (5.5)	126 (5.7)
Income			
Less than $15K	119 (3.5)	50 (2.4)	54 (2.5)
$15K - $30K	270 (7.9)	127 (6.0)	135 (6.1)
$30K - $50K	441 (13.0)	252 (11.9)	257 (11.7)
$50K - $80K	700 (20.6)	440 (20.8)	445 (20.2)
$80K - $150K	923 (27.2)	631 (30.0)	662 (30.1)
More than $150K	572 (16.8)	401 (19.0)	423 (19.2)
Missing	373 (11.0)	211(10.0)	221 (10.1)
Diagnosed Depression	670 (19.7)	485 (23.0)	501 (22.8)
Chronic Conditions			
zero	1,559 (.46)	578 (27.4)	445 (20.2)
one	1,837 (.54)	957 (45.3)	878 (39.9)
MCC	0	577 (27.3)	874 (39.7)
Existing Chronic Conditions			
Arthritis	633 (18.6)	-	-
Hypertension	777 (22.9)	-	-
Pulmonary	216 (6.4)	-	-
Heart Disease	123 (3.6)	-	-
Diabetes	88 (2.6)	-	-
Onset of Chronic Conditions			
Arthritis	-	492 (23.3)	288 (13.1)
Hypertension	-	282 (13.4)	196 (8.9)
Pulmonary	-	125 (5.9)	111 (5.0)
Heart Disease	-	120 (5.7)	132 (.6.0)
Diabetes	-	111 (5.3)	80 (3.6)

Results of the multilevel models examining change in depressive symptoms over time in this sample are presented in Table 2. Model A, unadjusted linear change in depressive symptoms over time, showed there was a significant increase in depressive symptoms over time (*β* = 0.41, *p* < .0001). Model B, adding demographic covariates revealed that age (*β* = −0.06, *p* < .0001) and income (*β* = −0.95, *p* < .0001) had significant negative associations with depressive symptoms and that gender (female) is positively associated (*β* = 0.42, *p* < .001) with depressive symptoms. Race (African American) was not significantly associated with depressive symptoms (*β* = −.033, *p* > .05). The positive relationship between time and depressive symptoms remained after accounting for the demographic characteristics (*β* = 0.57, *p* < .0001).

**Table 2.** Multilevel Models Predicting Depressive Symptoms Over Time

	Model A	Model B	Model C	Model D	Model E
	β *(SE*)	β *(SE*)	β *(SE*)	β *(SE*)	β *(SE*)
Intercept	4.51 (0.10)	11.79 (0.81)	9.66 (0.77)	9.77 (0.77)	10.9 (0.80)
Wave (Time)	0.41 (0.03)***	0.57 (0.05)***	0.53 (0.04)***	0.53 (0.04)***	0.82 (0.08)***
Age		−0.061 (0.01)***	−0.040 (0.01)***	−0.044 (0.01)***	−0.06 (0.012)***
Gender		0.42 (0.16)**	0.02 (0.15)	0.02 (0.15)	0.10 (0.16)
Income		−0.95 (0.06)***	−0.85 (0.06)***	−0.85 (0.06)***	−0.97 (0.06)***
Race (African American)		−0.33 (0.30)	0.06 (0.28)	0.064 (0.28)	0.04 (0.29)
Diagnosed Depression			3.88 (0.19)***	3.85 (0.19)***	3.89 (0.19)***
Onset MCC				0.96 (0.25)***	0.58 (0.27)*
Onset Arthritis					0.79 (0.19)***
Onset Hypertension					0.24 (0.19)
Onset Heart Disease					0.85 (0.32)**
Onset Diabetes					0.86 (0.36)*
Onset Pulmonary Disease					0.69 (0.28)*
Fit Indices					
-2 Log Likelihood	45,084.0	40,290.6	39,839.2	39,824.4	28,415.5
AIC^a^	45,092	40,306.6	39,857.2	39,844.4	28,445.5
BIC^a^	45,117.8	40,354.7	39,911.4	39,904.5	28,535.7
Chi-Square	1,598.25	1,294.21	1,076.79	1,060.43	510.0

*Note*: AIC = Akaike information criterion; BIC = Bayesian information criterion; MCC = Multiple chronic condition.

*N* = 3,396 baseline *p < .05, **p < .01, ***p < .001. ^a^Smaller is better.

Model C, adding diagnosed depression finds that age (*β* = −0.04, *p* < .0001) and income (*β* = −0.85, *p* < .0001) remained significantly negatively associated with depressive symptoms. Gender (female) (*β* = 0.02, *p* > .05) and race (African American) were not associated with depressive symptoms (*β* = .06, *p* > .05). Note that the associations of depressive symptoms with covariates in subsequent models remained similar to Model C. Individuals with a lifetime diagnosis of depression reported higher levels of depressive symptoms (*β* = 3.88, *p* < .0001). Time remained significantly positively associated with depressive symptoms (*β* = 0.53, *p* < .0001).

Model D finds that people who transitioned to having MCCs had higher levels of depressive symptoms (*β* = 0.96, *p* < .0001) than people who did not have MCCs. Individuals with a lifetime diagnosis of depression reported higher levels of depressive symptoms (*β* = 3.88, *p* < .0001). Time remained significantly positively associated with depressive symptoms (*β* = 0.53, *p* < .0001).

Model E revealed that accounting for the transition to MCC, the diagnosis of arthritis (*β* = 0.79, *p* < .0001; Cohen’s *D* = .19), heart disease (*β* = 0.85, *p* < .01; Cohen’s *D* = .26), diabetes (*β* = 0.86, *p* < .05; Cohen’s *D* = .24) and pulmonary disease (*β* = 0.69, *p* < .05; Cohen’s *D* = .22) each had independent, significant positive associations with depressive symptoms. Hypertension diagnosis was not significantly associated with depressive symptons (*β* = 0.24, *p* > .05). Diagnosed depression (*β* = 3.89, *p* < .0001; Cohen’s *D* = .62) and time (*β* = 0.82, *p* < .0001; Cohen’s *D* = .20) remained significantly positively associated with depressive symptoms. The fit indices reveal increasingly better fit, with the final model far superior to the previous ones.

## Discussion

This study prospectively examined the impact that transitioning to having multiple chronic conditions has on depressive symptoms in older adults, controlling for a lifetime diagnosis of depression. The magnitude of the association between lifetime diagnosis of depression and trajectory of depressive symptoms was the largest of any indicator in the models and demonstrates that it is critical to include this in analyses examining chronic illness and depressive symptoms. Results indicate that MCC onset, that is transitioning from having no or a single chronic condition to having two or more chronic conditions, is associated with higher levels of depressive symptoms over time. Furthermore, the diagnosis of arthritis, diabetes, heart disease, and pulmonary disease, but not hypertension, are independently associated with higher levels of depressive symptoms. These findings expand our understanding of the association of MCCs and depressive symptoms in older adults and carry important implications for research and practice.

Prior research has examined the impact of chronic illness on health and well-being outcomes in older adults (i.e., functional ability, depression, mortality; (McCall, Parks, Smith, Pope, & Griggs, 2002; Rotella & Mannucci, 2013a; Tinetti et al., 2011). However, most of this work has focused on single conditions (Heyworth et al., 2009; Saarni et al., 2007) or a raw count of illnesses (Laditka & Laditka, 2016), and has failed to tease out the causal association between depressive symptoms and chronic illness in the context of MCCs. The unique contribution of our analysis is the finding that the onset of multiple chronic illnesses impacts the development of depressive symptoms at multiple time points. As individuals transition from having no or a single diagnosed chronic condition to having MCCs, they report more depressive symptoms. This transition may represent a shift in the demand of illness management on the older person which is taking a toll on mental well-being. The management of chronic disease is often onerous and complex, requiring patients to make adjustments in diet, exercise, medication, or other lifestyle characteristics (Hughes, McMurdo, & Guthrie, 2012). As an additional illness is diagnosed, a new self-management routine is expected of the individual to maintain health, which may burden the individual and bring down his or her mental health. For example, if a person has arthritis, he may be expected to take anti-inflammatory medications and shift exercise routines to an aquatic setting to lessen pressure on joints. If he then gets a diagnosis of diabetes, he would additionally be expected to actively monitor blood sugar levels, maintain an exercise regime, modify dietary intake, and take additional medication. Although depressive symptoms are known generally to decline with age (which we report), the added pressure to respond to another illness may cause one to report more feelings of depression seen here in a sample that was relatively free from illness at baseline. Alternately, the onset of MCCs may impact depressive symptoms through one’s beliefs about oneself. For example, MCC onset may cause a person to feel as though he is declining, which results in facing his sense of mortality and therefore increases depressive symptoms. Or, the symptoms of the illness itself (i.e., pain, discomfort) may predispose a person to feeling more depressed (Beekman et al., 2002). A next step in research is to determine the explanatory mechanism through which the onset of MCCs impacts depressive symptoms. However, clinically, findings are relevant here, as older individuals present at a doctor’s office, practitioners must be aware of the burden that diagnosis of an additional chronic illness has on a person’s mental health and well-being. Social emotional support may be required for an individual to maintain mental health and functioning when grappling with the diagnosis of an additional chronic illness.

Beyond the onset of MCC status impacting depressive symptoms globally, we also see specific independent effects of a similar magnitude of chronic illness diagnosis on depressive symptoms for arthritis, diabetes, heart disease, and pulmonary disease but not for hypertension. This finding indicates that the type of illness diagnosed in the context of MCCs is important, not just the count (Pruchno et al., 2016). Hypertension is relatively benign in its symptoms and presentation. As a result, diagnosis of hypertension may not impact a person’s sense of self or daily habits, resulting in the lack of association found here. Arthritis, diabetes, heart disease, and pulmonary disease on the other hand are likely to be linked to uncomfortable symptoms (i.e., pain), complex treatment regimens, or feelings of mortality. Clinicians should be particularly aware of the impact a diagnosis of each of these conditions may have on a patient’s mental health and well-being and be prepared to connect older individuals to additional psychosocial support to help manage negative feelings.

Ultimately, the experience of depressive symptoms within an older adult population is detrimental to an individual’s psychosocial and functional well-being (Katon, 2011). Depression is linked to mortality (Schulz et al., 2000) and may also be linked to the development of additional chronic illnesses (Hasan, Clavarino, Mamun, Doi, & Kairuz, 2013; Karakus & Patton, 2011; Rotella & Mannucci, 2013a). Knowing that the diagnosis of a second or third or even later chronic illness is associated with depressive symptoms is critical, as it argues for the need for additional treatment supports. Furthermore, when that additional condition is arthritis, diabetes, heart disease, or pulmonary disease, such care is particularly important. Practitioners should explore ways to provide preventative mental health services to older individuals as they develop physical chronic illnesses. Researchers should also further explore the casual mechanisms of this association.

This study is not without limitations. The data utilized here are from a representative panel of older adults in New Jersey, but suffer from attrition effects over time. The sample under-represents individuals of Hispanic origin, men, and individuals with lower levels of education. The data are also localized to the state of New Jersey. Additional research should examine the associations tested here within other representative samples of older adults. Second, analyses here are unable to consider the impact of each unique combination of illnesses (i.e., pulmonary disease-heart disease-arthritis vs pulmonary disease-heart disease-diabetes) due to the complexity of the illness experience and small cell sizes that would result from slicing the data in this way. Additional work should build on strategies such as those used by Magnan and colleagues (2015) and Sales and colleagues (2008) that examine concordant and discordant comorbidities in reference to a specific index illness (i.e., diabetes) over time. Third, findings are limited to examination of only five chronic illnesses; additional work should examine the impact of additional illnesses. Fourth, we did not have an indicator of the severity of each illness. Although our analyses accounted for some known variables associated with depressive symptoms, we did not account for other characteristics, including disability, pain, social support, and cognition. Future studies should address this shortcoming. Finally, we caution that respondents were asked to report on diagnosed conditions at each time of measurement, yet being diagnosed with a condition does not mean that the condition actually developed or had any worse consequences for the individual after it was reported. As such, we are bound by the limits of self-reports of diagnosed illnesses.

Overall, the findings presented here are strengthened by the use of a prospective empirical design to examine the time ordered impact of onset of MCCs on depressive symptoms in older adults. Results indicate a clear association between acquisition of MCCs and depressive symptoms, particularly with the diagnosis of arthritis, diabetes, heart disease, and pulmonary disease. Clinical care providers should take account of these findings, encouraging psychosocial supports for older adults who develop MCCs to minimize the negative psychological impact of illness diagnosis. Research should further explore the mechanisms through which diagnosed illnesses act as stressful life events and impact depressive symptoms to inform practitioners. For example, work should determine whether diagnosis simply changes the expectations of the person or perception of others (Aneshensel, 1992; Bury, 1982) and has a resultant impact on psychosocial well-being (i.e., feelings of loss of independence or control over one’s life) or if diagnosis and/or changes in health behaviors (Newman et al., 2004) impact organic brain functioning (i.e., compromised brain functioning) which ultimately result in depression (Williamson et al., 2000). Understanding the lived experience of older individuals with MCCs is critical to ultimately supporting successful aging.

## Funding

The UMDNJ-SOM provided funding that enabled establishment of the ORANJ BOWL (“Ongoing Research on Aging in New Jersey – Bettering Opportunities for Wellness in Life”) research panel and collection of baseline data. The UMDNJ Foundation provided funding Time 3 data collection. Funding from the Assistant Secretary for Preparedness and Response (1 HITEP130008-01-00) and the Rockefeller Foundation (2012_RLC 304; PI: George Bonanno) supported Time 4 data collection.

## Conflict of Interest

None reported.
